# Brain-derived neurotrophic factor (BDNF): an effect biomarker of neurodevelopment in human biomonitoring programs

**DOI:** 10.3389/ftox.2023.1319788

**Published:** 2024-01-10

**Authors:** Andrea Rodríguez-Carrillo, Veerle J. Verheyen, Alexander L. N. Van Nuijs, Mariana F. Fernández, Sylvie Remy

**Affiliations:** ^1^ VITO Health, Flemish Institute for Technological Research (VITO), Mol, Belgium; ^2^ Toxicological Centre, University of Antwerp, Universiteitsplein, Wilrijk, Belgium; ^3^ Biomedical Research Center and School of Medicine, Instituto de Investigación Biosanitaria de Granada (ibs.GRANADA), Consortium for Biomedical Research in Epidemiology and Public Health (CIBERESP), University of Granada, Granada, Spain

**Keywords:** brain-derived neurotrophic factor, neurodevelopment, human biomonitoring, effect biomarkers; endocrine disruptors, neurodevelopmental toxicity

## Abstract

The present narrative review summarizes recent findings focusing on the role of brain-derived neurotrophic factor (BDNF) as a biomarker of effect for neurodevelopmental alterations during adolescence, based on health effects of exposure to environmental chemical pollutants. To this end, information was gathered from the PubMed database and the results obtained in the European project Human Biomonitoring for Europe (HBM4EU), in which BDNF was measured at two levels of biological organization: total BDNF protein (serum) and *BDNF* gene DNA methylation (whole blood) levels. The obtained information is organized as follows. First, human biomonitoring, biomarkers of effect and the current state of the art on neurodevelopmental alterations in the population are presented. Second, BDNF secretion and mechanisms of action are briefly explained. Third, previous studies using BDNF as an effect biomarker were consulted in PubMed database and summarized. Finally, the impact of bisphenol A (BPA), metals, and non-persistent pesticide metabolites on BDNF secretion patterns and its mediation role with behavioral outcomes are addressed and discussed. These findings were obtained from three pilot studies conducted in HBM4EU project. Published findings suggested that exposure to some chemical pollutants such as fine particle matter (PM), PFAS, heavy metals, bisphenols, and non-persistent pesticides may alter circulating BDNF levels in healthy population. Therefore, BDNF could be used as a valuable effect biomarker to investigate developmental neurotoxicity of some chemical pollutants.

## 1 Introduction

### 1.1 Biomarkers of effect and their use in human biomonitoring (HBM) programs

Human biomonitoring (HBM) programs are key tools for characterizing internal exposure to chemical contaminants in a population (Ganzleben et al., 2017; Ougier et al., 2021). Established HBM studies showed that humans are exposed to hundreds of natural and man-made chemicals through their daily life activities and that the chemicals are taken in the body. Moreover, collecting health information from the study subjects allows to investigate the link between internal exposure levels and adverse health effects (Colles et al., 2021). In this regard, our understanding of these relationships is increasing due to advancements in molecular knowledge, which allows the identification of the events occurred between the exposure and the effect (Colles et al., 2021; Ougier et al., 2021).

According to the World Health Organization (WHO), effect biomarkers are “*measurable biochemical, physiologic, behavioral, or other alteration in an organism that, depending on the magnitude, can be recognized as associated with an established or possible health impairment or disease*” (NRC, 2006). They provide information on a physiological system, which means that they may inform on the alterations leading to a particular health outcome. An ideal effect biomarker should be measured non-invasively and reflect changes associated with a disease in the target tissue, which makes them of high value, especially for those physiological systems with limited accessibility such as the brain (Aronson and Ferner, 2017; Rodriguez-Carrillo, 2022; Rodríguez-Carrillo et al., 2023).

Effect biomarkers have sparked a revolution in both epidemiology and toxicology substantially bolstering the evidence of a causal link between chemical exposure and the likelihood of adverse effects, particularly during the early stages of a disease (DeCaprio, 1997; Schisterman and Albert, 2012; Rodríguez-Carrillo et al., 2023). This approach marks a significant advancement compared to traditional epidemiological, toxicological, or analytical techniques, which lacked the necessary sensitivity to detect intermediate endpoints between exposure and clinical disease.

Effect biomarkers can be categorized based on the time course of contaminant-organism interactions into biomarkers of early and late biological effects (NRC, 2006). Biomarkers of early biological effects report initial effects after the biologically effective dose of the contaminant enters the human body (Mustieles et al., 2020). This category can include sister chromatid exchange, nuclear receptors activities, or epigenetic markers such as histone modification or DNA methylation. Biomarkers of late effects, show changes in structure or function at both the micro and macro levels (Baken et al., 2019; Mustieles et al., 2020). This broad group encompasses biomarkers ranging from biochemical effect biomarkers measured in biological matrices such as 8-hydroxy-2-deoxy-guanosine (8-OHdG) in urine, or hormones in serum, to changes at macromolecular or structural levels, such as anogenital distance (AGD), structural changes by magnetic resonance images (MRI), or functional neural effects by behavioral tests. Finally, biomarkers of genetic susceptibility enclose those who may determine the sensitivity of individuals to chemical exposure, i.e., single nuclear polymorphisms (SNP) (Kelly and Vineis, 2014). An overview of effect biomarkers used for human biomonitoring is provided in (Rodríguez-Carrillo et al., 2023).

### 1.2 Critical windows of neurodevelopment

Brain development commences about 3 weeks after conception, when the neural tube begins to take shape, and ends in early adulthood (about 25 years) (Stiles and Jernigan, 2010). When the embryonic period ends (gestational week 10) the initial architecture of the neural system is established, forming the so-called *connectome* (Stiles and Jernigan, 2010; Ardesch et al., 2019). After the age of 2, the brain undergoes synaptic pruning, a process in which inefficient connections are eliminated to enhance overall performance (Konkel, 2018). By the age of 6, the brain reaches approximately 90% of its adult volume (Stiles and Jernigan, 2010; Konkel, 2018; Ardesch et al., 2019).

During adolescence, individuals need to develop social-communicative skills, appropriate reproductive behaviors, and adequate responses to anxiety and affective states in line with their age and sex (Spear, 2000; Gore et al., 2018). The neural mechanisms underlying these changes are complex, involving a network of diverse brain areas interacting with each other and mediating specific aspects of behavioral functioning (Gore et al., 2018). In this context, two primary neural processes are significant: dendritic spine turnover and inhibitory neurotransmission (Pfeifer and Allen, 2021). A higher turnover of dendritic spines contributes to increased synaptic plasticity, thus facilitating experience-dependent learning. Additionally, increased inhibition of neurotransmission can be particularly beneficial for developing appropriate social behavior (Pfeifer and Allen, 2021).

During the last decades, there has been an increase in cases of attention deficit hyperactivity disorders (ADHD), autism spectrum disorders (ASD), and subclinical brain functioning impairments in children and adolescents, which can at least be partly attributed to chemical pollutants exposure (Grandjean and Landrigan, 2006; Grandjean and Landrigan, 2014). This situation has been referred to as the “silent pandemic of neurodevelopmental toxicity” (Grandjean and Landrigan, 2014). Moreover, there has been a rising incidence of mental health disorders among adolescents, such as social anxiety, depression, or eating disorders (Supke et al., 2021) for which exposure to chemical pollutants has been suggested to potentially amplify these effects (Bjørling-Poulsen et al., 2008; Bouchard et al., 2010; Shoaff et al., 2020; Pfeifer and Allen, 2021; Supke et al., 2021). However, the neurological impact of chemical pollutants exposure during adolescence remains relatively understudied, and there is still limited knowledge in this area.

## 2 BDNF, a molecular marker of brain development

### 2.1 Mechanisms implicated in the secretion, activation, and activity of BDNF in brain development

Brain-derived neurotrophic factor (BDNF) was discovered in 1982 and isolated from a pig’s brain in 1987. It is a member of the neurotrophin family and is expressed throughout the brain, with higher expression in specific regions such as the cerebral cortex, cerebellum, hippocampus, and amygdala (Bilgiç et al., 2017; Notaras and Buuse, 2018). BDNF plays a crucial role in various neurological processes involved in learning, memory, and cognitive functioning, *e.g.,* synaptic plasticity, neuroprotection, modulation of synaptic interactions, and the regulation of neurons and glia (Binder and Scharfman, 2004; Kowiański et al., 2018).

Moreover, BDNF is involved in the regulation of the neuroendocrine hypothalamus-pituitary-adrenocortical (HPA) axis (Naert et al., 2011). This axis, with the hormone cortisol as its main effector, is a crucial stress response system; the brain itself is also a target of stress-related hormones (McEwen, 2013). Research has demonstrated the relationship between stress and the expression of BDNF in many critical brain regions (Miao et al., 2020). Experimental and human studies indicate that chronic stress during adolescence may be associated with neurobehavioral alterations and cognitive disorders later in life (Sheth et al., 2017).

The diversity of BDNF functions arises from its ability to up or downregulate various signaling pathways due to its distinct synthesis pattern in which different biologically active isoforms interact with specific receptors (Bathina and Das, 2015). Thus, the biologically inactive precursor form of BDNF, pre-pro-BDNF, is secreted from the endoplasmic reticulum. The pre-region is cleaved in the Golgi apparatus, forming the immature isoform, pro-BDNF, which is biologically active and possesses an N-terminal pro-domain. Pro-BDNF is further cleaved to form its mature isoform, m-BDNF, which is also biologically active and contains a C-terminal mature domain (Autry and Monteggia, 2012; Kowiański et al., 2018). Both BDNF isoforms are secreted into the extracellular space upon membrane depolarization and often exhibit opposite biological activities due to their binding-receptors preferences (Reichardt, 2006; Kowiański et al., 2018).

Mature BDNF, m-BDNF, interacts with the tyrosine kinase B (TrkB) receptor, which is part of the tropomyosin-related kinase (Trk) family. This interaction triggers several cascades of mechanisms that lead to neuronal antiapoptotic and prosurvival activities, as well as enhancement of dendritic growth and branching, which are critical for the healthy functioning and development of neurons (Bathina and Das, 2015; Kowiański et al., 2018). The activation of phosphorylated-TrkB receptors after m-BDNF-TrkB binding activates different enzymes, including phosphatidylinositol 3-kinase (PI3K), mitogen-activated protein kinase (MAPK), guanosine triphosphate hydrolases (GTPases) from the Ras homolog (Rho) gene family, and phospholipase C-γ (PLC-γ); which initiates distinct signaling cascades, leading to specific cellular functions (Reichardt, 2006; Bathina and Das, 2015; Kowiański et al., 2018). MAPK is crucial for the activation of extracellular-signal-regulated kinase 1/2 (ERK 1/2) and cyclic adenosine monophosphate (cAMP) response element-binding protein (CREB) which play important roles in cytoskeleton protein synthesis, early response gene expression, branching, and dendritic growth in hippocampal neurons (Bathina and Das, 2015; Arévalo and Deogracias, 2023). The PI3K/Akt pathway modulates synaptic plasticity that depends on N-methyl-D-aspartate receptors (NMDAR), while the PI3K/Akt/mTOR pathway enhances dendritic growth and branching by regulating protein synthesis. PLC-γ activates the kinase C (PKC)-dependent pathway, which increases synaptic plasticity. Last, the GTPases cascade also contributes to dendritic growth by promoting the synthesis of microtubules and actin (Kowiański et al., 2018).

The precursor form of BDNF, pro-BDNF interacts with a specific receptor called p75 neurotrophin receptor (p75NTR), forming the pro-BDNF/p75NTR/sortilin complex. When pro-BDNF binds to its receptor, it triggers signaling pathways that influence the fate of neurons in various brain regions, either promoting cell death or supporting cell survival. It is worth noting that elevated levels of pro-BDNF have been reported to lead to neuronal cell elimination rather than promoting neuronal cell survival (Teng et al., 2005; Notaras and Buuse, 2018). The activation of the c-Jun amino-terminal kinase (JNK) signaling cascade initiates neuronal apoptosis, which results in neuronal cell death. In contrast, the activation of nuclear receptor kappa B (NR-κB) promotes processes that support survival and the maintenance of an optimal number of neuronal cells. Finally, the Ras homolog gene family member A (RhoA) cascade regulates the growth cone of neurons, a crucial structure involved in neurite outgrowth and guidance (Reichardt, 2006; Kowiański et al., 2018; Arévalo and Deogracias, 2023).

### 2.2 Evidence of the use of circulating BDNF levels to assess chemicals exposure effects

A narrative review was conducted to know the available epidemiological evidence regarding the use of BDNF as effect biomarker for neurodevelopment. Table 1 shows the search strategy performed in the PubMed database. Four search strategies were used to cover as much information as possible. Initially, 331 preliminary publications we found. From these, 48 were selected after title and abstract screening, and only 22 studies met the inclusion criteria: i) healthy population exposed to environmental chemical contaminants, ii) case-control/cohort studies reporting BDNF protein levels, BDNF gene methylation, or SNPs of the BDNF gene, iii) articles published within 10 years, iv) free available full-text article and written in English, and v) scoring ≥7 points in the New-Castle Ottawa Scale (NOS). The NOS is the ‘star-system’ to evaluate the quality of epidemiological (cohort and case-control) studies based on three main characteristics: the selection of the study groups; the comparability of the groups; and the ascertainment of either the studied exposure or the outcome, with a maximum scoring of 9 points. Supplementary Table S1 shows the scoring results per study.

**TABLE 1:** Research strategy to find and select epidemiological studies assessing environmental contaminants and BDNF levels.

No. Search	Date	Database	Search terms	Filters	Results	Title/Abstract screening	Selected	Observations
1	31/08/2023	PubMed	(BNDF AND brain derived neurotrophic factor) AND pollutants	10 years, Full-text, and Humans	43	18	13	5 studies were excluded: 2 pilot studies of the INMA-Granada cohort^a^; 1 article written in Chinese; and 2 review articles
2	31/08/2023	PubMed	(Brain derived neurotrophic factor OR BDNF) AND (Environmental chemicals)		65	6	1	5 studies were excluded: 3 repeated articles (search no.2); 1 without BDNF values; and 1 revision
3	31/08/2023	PubMed	BDNF AND (endocrine disruptor OR endocrine disrupter OR endocrine disrupting chemical)		17	1	1	
4	31/08/2023	PubMed	BDNF AND (BPA OR Phthalates OR PAH OR Cd OR benzophenones OR PBDE OR Cr OR PFOS OR PFOA OR Acrylamide OR As OR Pb OR Hg OR Mycotoxins OR pesticides)		206	23	7	16 studies were excluded: 1 repeated study; 1 study without chemical exposure levels; 14 already selected in searches no. 1 and 2
				Total	331	48	22	

^a^ Studies from INMA-Granada cohort were not included since they were results from the HBM4EU, project. Those articles are included and discussed in section 3.

Studies received ≥7 points in the NOS (Supplementary Table S1), thus being of optimal quality. Regarding design, 11 of the 22 selected studies had a cross-sectional design, 8 were longitudinal, and 3 were case-control studies (Table 2). The total number of participants in all studies was 7,693, of whom 54.74% were newborns or mother-son pairs, including pregnant women, 30.09% children, and 15.17% adults (Supplementary Table S2). The included studies evaluated the effects of the following chemical pollutants: acrylamide, polycyclic aromatic hydrocarbons (PAHs); per- and polyfluoroalkyl substances (PFAS); particle matter with a diameter ≤1, 2.5, 10 μm (PM1, PM2.5, PM10, respectively); nitrogen dioxide (NO_2_); and metals: aluminium (Al); arsenic (As); cadmium (Cd); mercury (Hg); methylmercury (MeHg); manganese (Mn); lead (Pb); vanadium V); zinc (Zn) (Table 2). Further, 14 of the 24 studies measured total BDNF, which refers to the measurement of both isoforms, pro- and mature BDNF, in serum, plasma, or cord blood; 4 studies evaluated the single nuclear polymorphisms of BDNF (SNP) in oral swab samples or cord blood; 3 studies the mRNA or DNA methylation of the BDNF gene in cord blood; and 1 study the mature isoform of BDNF (Table 2).

**TABLE 2:** Epidemiological studies addressing the associations between diverse chemical pollutants and BDNF levels published during the last 10 years (n = 22). Studies are displayed in order according to the chemical exposure tested.

Study					Exposure assessment		BDNF		Results	NOS score
No. Study	First author, year	Design	Objective	Size and characteristics of population	Exposure	Matrix	Type	Matrix		
**1**	Wang et al. (2023)	Longitudinal	To investigate the association of maternal PM2.5 concentrations with fetal BDNF levels at birth	711 mother-son pairs	Ambient fine particulate matter PM2.5	Estimated using a random forest model^a^	Total BDNF	Umbilical cord blood	One natural log unit (ln-unit) increase in maternal PM2.5 levels during the second trimester was significantly associated with β = -0.20 (95% CI: 0.36,-0.05) ln-unit decrease in BDNF levels at birth	9
**2**	Huang et al. (2022)	Longitudinal	To study the association between air pollutants and methylation of peripheral BDNF promoters	34 healthy adults (69.2 ± 5.4 years)	PM1, PM2.5, PM10, and NO_2_	Satellite basedrandom forest	DNA methylation BDNF gene at exon IV	Whole blood	The percent change of average methylation level at the 95th percentile of NO2 against the threshold concentration was 43.25% (95%CI: 13.10%, 73.40%), and that of CpG2 methylation at the 95th percentile of PM1 was 128.29% (95%CI: 43.27%, 213.31%)	9
**3**	Yu et al. (2021)	Longitudinal	To investigate the association between prenatal PFAS and cord blood BDNF levels	725 mother-child pairs	PFAS	Maternal plasma	Total BDNF	Cord blood	A significant positive association was observed between PFHxS and BDNF levels [β = 1,285 (95% CI: 453, 2,118, *p* = 0.003)]. No association was observed with other PFAS congeners or the PFAS mixture on BDNF levels	8
**4**	Zhou et al. (2019b)	Longitudinal	To investigate prenatal vanadium V) concentrations and fetal growth	227 mother-child pairs	V	Maternal serum	Total BDNF	Cord blood	No statistical associations were found	9
**5**	Wang et al. (2016a)	Longitudinal	To explore the association between prenatal Cd concentrations and infants’ developmental quotients (DQs) at 12 months and the role of BDNF in prenatal cadmium-induced neurodevelopmental deficits	300 mother-child pairs	Cd	Maternal blood	Total BDNF	Cord blood	A 10-fold increase in maternal Cd concentrations were associated with a 4.31 (95% CI, −8.05,-0.57) point decrease in BDNF levels	8
**6**	Saenen et al. (2015)	Longitudinal	To assess *in utero* exposure to fine particle air pollution and placental expression of genes implicated in neural development	90 mother-child pairs	PM2.5	Spatiotemporal Interpolation method (kriging) that uses land cover data obtained from satellite images and monitoring stations	BDNF gene expression	Placental tissue	A 5-μg/m^3^ increase in residential PM2.5 exposure during the first trimester of pregnancy was associated with a 15.9% decrease [95% confidence interval (CI): −28.7, −3.2%] in expression of placental BDNF levels. However, no statistical association was found during the second and third trimesters	9
**7**	Song et al. (2023)	Cross-sectional	To study the acute effect of NO_2_ exposure on nervous system damage biomarker levels in healthy older adults	34 healthy adults (63.6 ± 5.7 years)	Ambient nitrogen dioxide (NO_2_)	Fixed site monitor	Total BDNF	Serum	Each 10 μg/m^3^ increase in NO_2_ concentration was associated with 49% increase (95% CI, 2.96%) in BDNF levels	7
**8**	Song et al. (2022)	Cross-sectional	To study the association between the overall effect of PM 2.5 and biomarkers of neurological damage, particularly BDNF.	34 healthy adults (63.6 ± 5.70 years)	Ambient fine particulate matter (PM2.5)	24 h mean concentrationof PM2.5 collected from an air quality monitoring station	Total BDNF	Serum	Each 10 μg/m^3^ increase in PM2.5 concentration was associated with 2.09% (95% CI, 39.3; 76.5%) of BDNF levels	7
**9**	Zhou et al. (2021)	Cross-sectional	To investigate the effects of low-level Hg exposure on serum BDNF levels in children	541 newborns (69.3 ± 6.56 months)	Hg	Blood	Total BDNF	Serum	Significant positive association between blood mercury concentrations and serum BDNF [β = 4.91, 95% CI = 1.20,8.63]. The association was attenuated after adjusting for dietary and passive smoking, although remained significant in girls	8
**10**	Malavika et al. (2021)	Cross-sectional	To evaluate the association of blood lead levels with neurobehavioral alterations and changes in BDNF expression	72 children (9–15 years)	Pb	Blood	BDNF mRNA and Total BDNF	Whole blood and serum, respectively	Significant positive association between Pb concentrations and serum BDNF levels (r = 0.25, *p*-value = 0.04) and with BDNF mRNA expression (r = 0.28, *p*-value = 0.01) using Pearson’s correlation test	7
**11**	Hogervorst et al. (2021)	Cross-sectional	To study the associations between acrylamide biomarkers and markers related to fetal growth	443 newborns	Acrylamide, glycidamide hemoglobin adduct	Cord blood	Total BDNF	Cord plasma	No statistical associations were found	8
**12**	Zhou et al. (2019a)	Cross-sectional	To assess the association between blood Pb, Hg, Al and Mn levels and serum BDNF concentrations in 17pre-school children	561 pre-school children	Pb, Hg, Al and Mn	Blood	Total BDNF	Serum	Blood Pb concentrations were significantly negatively associated with serum BDNF concentrations in all the subjects (β, 95%CI: −4.83 [-9.40,-0.25]). When dichotomizing according to sex, the association remained significant for boys (−7.32 [-13.65,-0.99) but not for girls	8
**13**	Zaw and Taneepanichskul (2019)	Cross-sectional	To assess the association of maternal blood heavy metal concentrations and BDNF during the first trimester of pregnancy	108 pregnant women	Pb, Hg, Cd, and As	Blood	Total BDNF	Plasma	High blood total As concentrations had 2.6-fold increased odds (OR = 2.60, 95% CI: 1.18, 5.75) of low plasma BDNF levels as compared with low blood total arsenic group. No significant association between BDNF and Pb, Hg and Cd was found	8
**14**	Karim et al. (2019)	Cross-sectional	To assess the associations between As concentrations and adult cognitive impairment using the Mini-Mental State Examination (MMSE) and the serum levels of BDNF.	693 adult (18–60 years)	As	Drinking water and nails	Total BDNF	Serum	Each IQR increase in water, hair and nail As concentrations were associated with lower serum BDNF levels [(β, 95%CI: −0.44 (−0.61, −0.27); −0.22 (−0.38, −0.06); and −0.31 (−0.49, −0.13), respectively]	8
**15**	Zhang et al. (2017)	Cross-sectional	To assess the effect of Pb concentrations on child olfactory memory, including BDNF levels	118 children (4–7 years)	Pb	Blood	Total BDNF	Serum	Blood Pb concentrations were significantly associated with serum BDNF levels: 0.68 95%CI (0.21,1.15)	7
**16**	Perera et al. (2015)	Cross-sectional	To assess the association between PAHs DNA adducts, mature BDNF in newborns and their cognitive development at 2 years	505 newborns	PAHs	Cord blood	Mature BDNF	Cord plasma	A significant association between PAHs DNA adducts and lower mature BDNF levels (β = −0.11, *p*-value = 0.02) was found	8
**17**	Yang et al. (2016)	Case-control	To examine the differences in the concentrations of 280 plasma cytokines between 8 coke-oven workers and 16 community residents exposed to PAHs	24 adults: 8 exposed workers (50.4 ± 6.4 years) and 16 controls (48.3 ± 4.3 years)	PAHs	Urine	Total BDNF	Serum	Each increase in OH-PAHs IQR was associated >16% lower BDNF (*p* < 0.05)	9
**18**	Zou et al. (2014)	Case-control	To assess the effect of occupational exposure to Mn on plasma BDNF levels	248 workers (40.2 years) 100 controls (35.7 years)	Mn	Air samples	Total BDNF	Plasma	A significant difference in plasma BDNF levels between the control group (288.7 ± 181.7 pg/mL) and the three exposed subgroups (223.4 ± 178.3 pg/mL, 178.2 ± 138.1 pg/mL, 127.5 ± 99.8 pg/mL for workers in low, intermediate, and high-exposure groups, respectively, was found. Furthermore, there was a linear trend between plasma BDNF levels and exposed groups	9

Epidemiologic studies assessing the exposure to certain environmental contaminants in the presence of BDNF single nuclear polymorphisms (SNPs)										
**19**	Sarzo et al. (2022)	Longitudinal	To examine the influence of different BDNF SNPs on the association between pre and postnatal Hg concentrations and children’s sexual development	413 children (9.1 ± 0.2 years)	Prenatal and postnatal Hg concentrations	Prenatal: umbilical cord blood; Postnatal: hair	BDNF SNPs: rs12273363, rs7934165, rs7103411, rs1100104, rs6265, and rs925946	Cord blood	Significant genetic interactions for rs11030104 and rs6265 and cord blood Hg concentration in male, and significant interactions between the SNPs rs7103411, rs11030104, rs7934165 and rs6265 and cord blood mercury in females	9
**20**	Julvez et al. (2013)	Longitudinal	To evaluate the role of SNP of BDNF in the associations between exposure to methylmercury (MeHg) and the cognitive function of children	843 children	MeHg	Cord blood	BDNF SNPs: rs2049046	Cord blood	No statistical associations were found	9
**21**	Lozano et al. (2021)	Cross-sectional	To explore the relationship between Hg concentrations at 9 years old and behaviour assessed at 9 and 11 years old and how BDNF SNP interact	Children at two follow-ups: 403 children (9 years) and 328 (11 years)	Total Hg (THg)	Hair	BDNF SNPs: rs7934165, rs6263, rs12273363, rs7103411	Cord blood	Some marginally statistically significant interactions (*p*-value <0.1) were observed between THg concentrations and the SNPs rs1519480 and rs7934165. Children with CC genotypes for rs1519480 and AA for rs7934165 obtained worse scores on externalizing problems (β, 95%CI: 1.13 [1.01, 1.26], 1.25 [1.05, 1.48], respectively) with increasing THg concentrations than the T and G allele carriers (β, 95%CI: 0.98 [0.87, 1.09] and 1.05 [0.93, 1.17], respectively)	8
**22**	Zhu et al. (2022)	Case-control	To examine whether the combined effect between metals exposure [including Pb, Hg, As, cadmium (Cd), zinc (Zn), manganese (Mn), V, and Cu] and BDNF polymorphisms was related to dyslexic risk	Children (9.7 ± 1.3 years): 238 dyslexic, 228 no dyslexia	Pb, Hg, As, Cd, Zn, Cu, Mn, and V	Urine	BDNF SNPs: rs6265, rs2883187, rs7124442	Oral swab samples	Significant associations of dyslexic risk with rs6265 polymorphisms of the BDNF gene were observed (OR = 1.99; 95% CI: 1.15,3.44). Furthermore, exposure to Cu could interact with rs6265 to increase the risk of dyslexia (*p* interaction = 0.045). High-Cu children with the rs6265 TT genotype were more likely to have dyslexia compared with low-Cu children carrying CC + CT genotypes (OR = 3.19; 95% CI: 1.38,7.39)	9

^a^ Estimation was performed by combining satellite aerosol optical depth (AOD) and spatiotemporal predictors; BDNF, brain-derived neurotrophic factor; IQR, interquartile range; SNPs, single nuclear polymorphisms; Total BDNF, mature and pro-BDNF, isoforms; PAHs, polycyclic aromatic hydrocarbons; PFAS:, perfluoroalkyl substances; PM1, PM2.5, PM10, particle matter with a diameter ≤1, 2.5, 10 μm respectively; NO_2_ nitrogen dioxide; Al, aluminium; As, arsenic; Cd, cadmium; Hg, mercury; MeHg, methylmercury; Mn, manganese; Pb, lead; V, vanadium; Zn, zinc New-Castle Ottawa Scale (NOS) score to evaluate the quality of the cohort and case-control selected studies.

Longitudinal studies addressing ambient air exposure found that PM2.5 during pregnancy was associated with lower total BDNF levels in newborns (Saenen et al., 2015; Wang et al., 2023), whereas cross-sectional studies focused on the old adult population (>60 years) found that NO_2_ was associated with higher serum BDNF levels (Song et al., 2022; Song et al., 2023). Exposure to PM1 and NO_2_ in older healthy adults was cross-sectionally associated with higher DNA methylation of the BDNF gene (Huang et al., 2022).

Regarding metal elements, studies on exposure to V and MeHg did not find any statistical association with serum BDNF levels or any interaction with single nuclear polymorphisms (SNPs) of the BDNF gene (Julvez et al., 2013; C. C; Zhou et al., 2019b). Interestingly, As exposure from various sources (e.g., hair, nail, air, and blood) was associated with decreased total plasma and serum BDNF levels in adults (18–60 years) including pregnant women (Karim et al., 2019; Zaw and Taneepanichskul, 2019). In the same line, prenatal blood Cd and occupational air Mn concentrations were associated with lower total serum BDNF levels in children and workers, respectively (Y. Wang et al., 2016b; Zou et al., 2014). Results in studies assessing blood Pb concentrations in children were less consistent: in two cross-sectional studies Pb concentrations were associated with higher BDNF gene expression and total serum BDNF levels (Zhang et al., 2017; Malavika et al., 2021), whereas Pb was cross-sectionally associated with lower total serum BDNF levels in preschool children (Zhou et al., 2019a). In the remaining studies, no further statistical associations were found (Zaw and Taneepanichskul, 2019; Zhu et al., 2022). Regarding SNPs, children with rs6265 exposed to Cu showed higher odds of having dyslexia (Zhu et al., 2022), and those with rs11030104, rs6265, rs1519480, and rs7934165 exposed to Hg showed an interaction for worse scoring in different behavioral scales, e.g., externalizing problems (Lozano et al., 2021; Zhou et al., 2021). Moreover, two cross-sectional studies assessing urinary PAHs concentrations in adults (Yang et al., 2016) and blood PAHs DNA adducts in newborns found associations with lower total BDNF levels (Perera et al., 2015). Finally, a longitudinal study assessing blood PFAS concentrations found increased BDNF levels in children (Yu et al., 2021), whereas a study assessing acrylamide exposure found no associations (Hogervorst et al., 2021).

It seems that some metals showed a consistent association with decreasing BDNF levels during childhood and adulthood, which could increase the odds of developing neurodevelopmental alterations. Air pollution with PM2.5 was associated with decreased BDNF concentrations during the prenatal period; therefore, a higher air quality should be considered as a recommendation during pregnancy. Increased BDNF due to fine PM exposure in adulthood could have different explanations. Since total BDNF instead of the pro-BDNF isoform was considered, it is difficult to conclude that the observed increase was due to pro-BDNF secretion, which would trigger the apoptotic pathways, thus leading to neurodegenerative processes (Kowiański et al., 2018). In this regard, further research is needed considering pro- and mature BDNF isoforms separately to have a clearer picture of the events between the exposure and the neurodevelopmental effect. PAHs exposure was related to decreased BDNF levels in two studies, which is in line with the overall trend of findings in epidemiological studies. However, the design and quantity of studies are not sufficient to reach any firm conclusions.

Moreover, the studies included in the present narrative review showed some limitations that may influence their observations, consequently, their results should be interpreted with caution. First, the study design: studies showing cross-sectional designs cannot infer causality, which is the most concerning limitation when interpreting their results. Therefore, longitudinal are preferred over cross-sectional studies. Second, population heterogeneities due to within-study differences in age, sex, and ethnicity, may influence observations, thus reducing the comparability of results. Third, the chosen window of susceptibility, since ND mechanisms differ greatly according to age. Fourth, the chosen methodology when measuring the exposure: repeated biospecimens per subject are encouraged for a better knowledge of the current exposure to certain chemical pollutants, especially non-persistent chemicals (Casas et al., 2018). However, the selected studies used a spot sample to ascertain the exposure concentrations, or by consulting external sources, thus increasing misclassification risk, and underestimating the observed effect. This may explain the lack of association between chemical pollutants and BDNF in some epidemiological studies. Therefore, there is a need for more and better-designed epidemiological studies addressing developmental neurotoxic effects concerning environmental pollutants exposure such as PAHs, PFAS, acrylamide, phenols, or non-persistent pesticides. Some recommendations in this regard would be first, to conduct longitudinal studies, thus being able to establish a causal exposure-effect relationship. Second, recruit enough participants to achieve adequate statistical power. Third, to assess the exposure to chemical pollutants by collecting repeated biospecimens, thus decreasing bias due to temporal or within-subject variability. Finally, to measure serum BDNF protein in a standardized manner to avoid a high inter-assay coefficient of variability when using immunosorbent assays, such as the one developed in HBM4EU (Fernández et al., 2021).

## 3 Discussion on the pilot studies assessing BDNF as a biomarker of neurotoxicity

As previously shown, alterations of this neurotrophin were also associated with longitudinal exposure to PAHs, Cd, and PM (Kalia et al., 2017; Saenen et al., 2016; Y; Wang et al., 2016a) in healthy children and with cross-sectional exposure to arsenic (As) in adults (Karim et al., 2019). Additionally, other studies found lower serum BDNF protein levels in patients with attention deficit hyperactivity disorder (ADHD), schizophrenia, bipolar disorder, major depressive disorder (MDD), and autism spectrum disorder (ASD) (Akay et al., 2018; Bakirhan et al., 2017; Francis et al., 2018; Jiang et al., 2017; S. et al., 2017). Further, alteration in its DNA methylation levels, assessed by measuring the CpG islands from the Exon IV of the *BDNF* gene, was shown to be associated with bipolar disorder and major depression (Fuchikami et al., 2011; Kundakovic et al., 2015). Moreover, the single nucleotide polymorphism Val66Met of the BDNF gene may highlight the susceptibility to develop certain neurological disorders (Zhao et al., 2017).

However, the mechanistic understanding of these associations is not fully addressed. Thus, within the Human Biomonitoring for Europe Initiative (HBM4EU), the first European project to set up an HBM program at the European level combining biomarkers of exposure and effect (Ganzleben et al., 2017; Ougier et al., 2021), BDNF was proposed as a candidate effect biomarker to better understand the link between exposure to chemical pollutants and changes in neurodevelopment (Rodríguez-Carrillo et al., 2023). Since neurological alterations may be particularly concerning during adolescence (Pfeifer and Allen, 2021), a series of pilot studies in adolescents with measurements of BDNF levels were developed (Rodriguez-Carrillo et al., 2022a; Rodriguez-Carrillo et al., 2022b; Mustieles et al., 2022).

The study participants were male adolescents (aged 15–17 years) from the longitudinal INMA-Granada (*Infancia y Medio Ambiente*–Environment and Childhood) cohort (Granada, Spain) (Castiello et al., 2020). The first study focused on assessing the relationships between bisphenol A (BPA) exposure, measured in urine when participants were 9–11 years old, with the behavioral function of participants in adolescence (15–17 years old), measured with the Child Behavior Check List (CBCL 6/18); taking into account both serum BDNF levels and BDNF gene methylation in six CpGs of Exon IV, also measured during adolescence. (Mustieles et al., 2022). The same approach was also made considering the exposure to some neurotoxic metals and non-persistent pesticide metabolite concentrations, respectively measured urine matrices during adolescence (Rodríguez-Carrillo et al., 2022b; Rodriguez-Carrillo et al., 2022b). More information regarding the study designs is available in Table 3.

**TABLE 3:** Characteristic of the pilot studies designs performed with adolescent males from the INMA-Granada cohort.

No. pilot study	References	Design	Measurement	Participants’age (years)	Matrix/Scale	Sample collection	Sample size
**No. 1**	Mustieles et al. (2022)	Longitudinal	BPA	9 to 11	Urine	First morning	130
			Total BDNF	15 to 17	Serum	Non-fasting	124
					Urine	First morning	124
			BDNF DNA methylation	15 to 17	Whole blood	Non-fasting	118
			Behavioral function	15 to 17	CBCL		130
**No. 2**	Rodríguez-Carrillo et al. (2022b)	Cross-sectional	As	15 to 17	Urine	First morning	125
			Cd				125
			Hg				125
			Pb				125
			total BDNF		Serum	Non-fasting	125
			BDNF DNA methylation		Whole blood	Non-fasting	113
			Behavioral function		CBCL		125
**No. 3**	Rodríguez-Carrillo et al. (2022a)	Cross-sectional	OPs: TCPy, IMPy, MDA, DETP, DEDTP	15 to 17	Urine	First morning	140
			PYR: 3-PBA, cis- trans-DCCA				
			Carbamates: 1-N				
			Dithiocarbamates: ETU				
			total BDNF		Serum	Non-fasting	130
			BDNF DNA methylation		Whole blood	Non-fasting	118
			Behavioral function		CBCL		130

CBCL, Child Behavioral Checklist 6/18; OP, organophosphate metabolites; TCPy, 3,5,6-trichloro-2-pyridinol; IMPy, 2-isopropyl-6-methyl-4-pyrimidinol; MDA, malathion diacid; DETP, dialkyl phosphates diethyl thiophosphate; DEDTP, diethyl dithiophosphate; PYR, pyrethroid metabolites; 3-PBA, 3-phenoxybenzoic acid; cis-trans-DCCA, dimethylcyclopropane carboxylic acid (sum of cis and trans isomers); 1-N, 1-naphthol; ETU, ethylene thiourea.

### 3.1 BDNF as a mediator for behavioral function in adolescents exposed to BPA during childhood

Pilot study no. One from the INMA-Granada cohort (Supplementary Table S2) found that higher childhood urinary BPA concentrations were longitudinally associated with increased behavior problems in adolescence, especially thought problems [0.76 (0.02.1.49)]. Childhood BPA exposure was also longitudinally associated with a higher percentage of DNA methylation at the promoter region IV of the BDNF gene measured in adolescence at CpG number 6 [β, 95% CI: 2.59 (0.31,4.87)]. However, no statistical associations were found for serum or urinary BDNF protein levels. Nevertheless, a mediation effect was found for DNA methylation of the BDNF gene at CpG6 [β, 95% CI: 0.23 (0.01,0.57)], which accounted for up to 34% of the effect between BPA and thought problems.

Although non-significant associations were found between childhood BPA and the protein isoform of BDNF measured in serum, statistically significant correlations with various CpGs of the BDNF gene were found. Moreover, the observed directions of the coefficients assessing serum BDNF-BDNF DNA methylation were according to expected, since the higher the DNA methylation, the lower the protein levels. Moreover, serum BDNF was negatively and significantly associated with various CBCL scales, which is biologically plausible (Rodríguez-Carrillo et al., 2022a; Rodríguez-Carrillo et al., 2022b). However, urinary BDNF showed the opposite trend: its association with BDNF DNA methylation was positive, which was not expected, also, it did not show any significant associations with any CBCL scales. This may be due to the secretion of BDNF by the bladder during muscle contraction in the micturition process (Ece et al., 2019). Urinary BDNF has been proven to be an optimal marker to detect overactive bladder diseases, as reported in children with enuresis (Ece et al., 2019; Morizawa et al., 2019). Although urinary BDNF may not be the ideal effect biomarker to address CNS alterations due to its secretion by kidneys and bladder tissues (Ece et al., 2019), several studies have highlighted its use as an indicator of visceral chronic pain such as Bladder Pain Syndrome/Interstitial Cystitis (BPS/IC) and Chronic Prostatitis/Chronic Pelvic Pain Syndrome. Thus, the use of urinary BDNF in HBM programs may be more suitable for chronic pain-related conditions.

**FIGURE 1:**
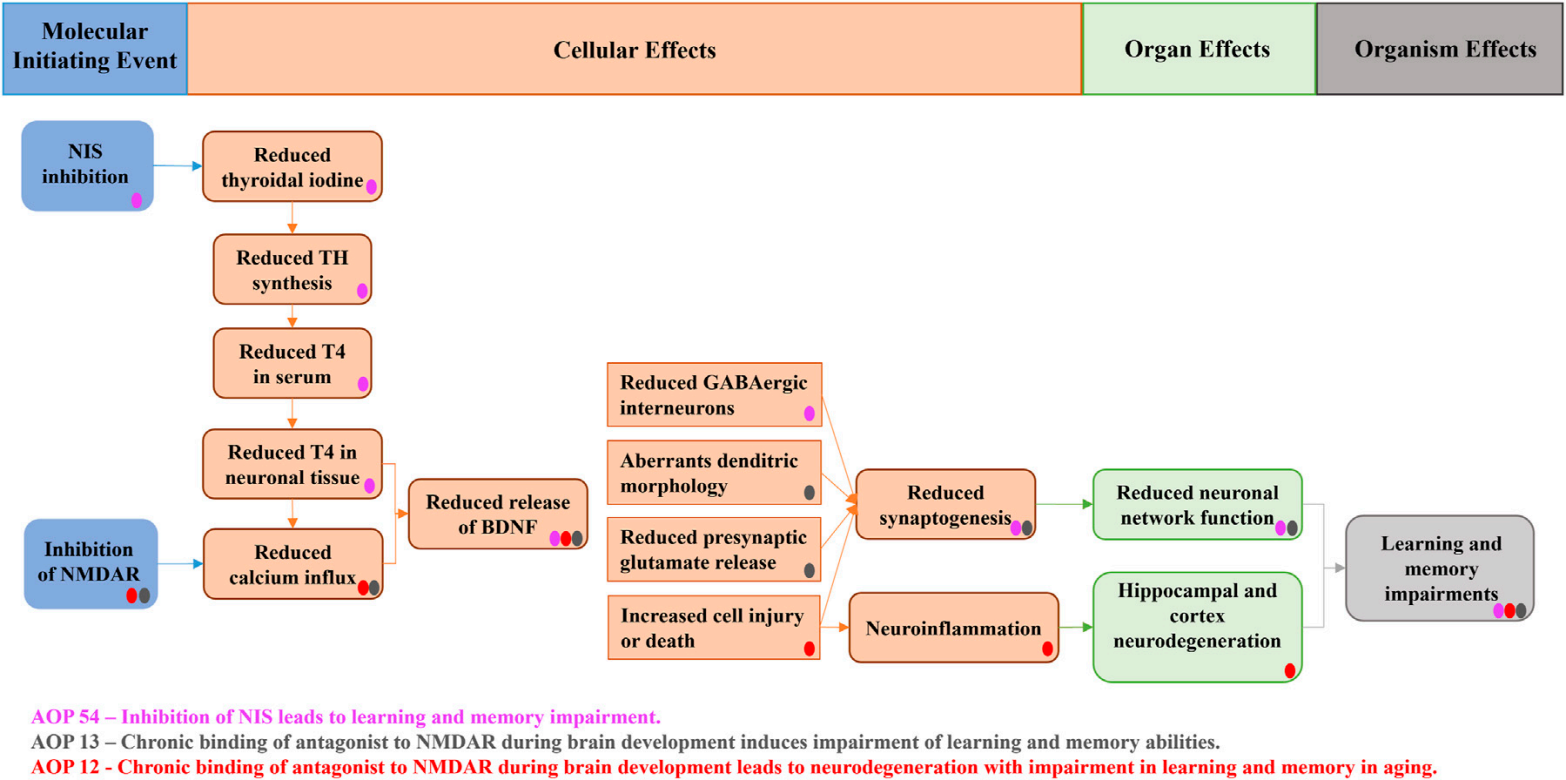
AOP network supporting the associations between childhood BPA, BDNF levels, and behavioral function in adolescent males. *Figure modified from* (Mustieles et al., 2020). *NIS: sodium/iodide symporter; NMDARs: N-methyl-D-aspartate receptors; TH: thyroid hormone; T4: thyroxine. Three completed AOPs available in the AOP wiki (AOP 12, 13, and 54) were converged in the current AOP network. Blue boxes represent molecular initiating events (MIEs), orange boxes key events (KEs) and green and grey boxes the resulting alteration in brain development. According to toxicological studies, BPA interacted with most MIE and KEs through the inhibition of NIS and NMDARs, thus leading to reduced hippocampal BDNF release and ultimately to impairments in learning and memory.*

As shown in Figure 1, there are some adverse outcome pathways (AOPs) that could support and explain these results. For instance, based on AOPs #12 and #13 available at the AOP wiki (https://aopwiki.org/), exposure to BPA (bisphenol A) is likely to decrease glutamate intake by inhibiting N-methyl-D-aspartate receptors (NMDAR) (Figure 1). Consequently, this inhibition would result in a decrease in calcium influx, leading to impaired activity of Calcium/calmodulin-dependent kinase II (CaMKII), a key factor in BDNF (brain-derived neurotrophic factor)-mediated neuroprotection, as demonstrated *in vitro* (Fan et al., 2006). Furthermore, the reduced calcium influx also affects the inactivation of cAMP response element-binding protein (CREB), with BDNF being a known target of CREB in rat models (Wang et al., 2016a). Accordingly, in animal studies, prenatal exposure to BPA has been associated with decreased expression of NMDAR subunits in the hippocampus and alterations in learning and memory cognitive domains in male pups of mice and rats (Tian et al., 2010; Xu et al., 2010; Wang et al., 2014), as well as in female and male mice exposed to BPA after birth (Jardim et al., 2017).

Moreover, in male mice, prenatal BPA exposure has been linked to reduced CREB phosphorylation in the hippocampus, resulting in decreased levels of NMDAR2B and BDNF mRNA (Kundakovic et al., 2015). These findings suggest that BPA exposure during critical developmental stages may have significant effects on brain function and cognition. Additionally, AOP #54 indicates that BPA may act as an inhibitor of the sodium/iodide symporter (NIS), as observed in a rat model (Wu et al., 2016). This inhibition could potentially lead to decreased mRNA levels of NIS and thyroid peroxidase (TPO) as reported by Silva et al. (2018). Consequently, this reduction in NIS activity would impair iodine uptake and hinder TPO activity, resulting in decreased levels of thyroid hormones (TH) and BDNF secretion. Various experimental studies have reported a decrease in thyroxine (T4) levels in pregnant ewes, their offspring, and aged mice exhibiting impaired cognitive function due to BPA exposure (Viguié et al., 2013; Jiang et al., 2016). However, Silva et al. (2018) observed increased T4 levels in rats exposed to BPA instead of a decrease. These findings highlight the complexity of BPA’s effects on thyroid hormone regulation and the need for further research to fully understand its impact on the endocrine system.

Although the information on the AOP network from Figure 1 gives promising insights, other mechanisms should also be considered. For instance, NMDAR can be regulated by hippocampal nuclear estrogen receptors (ER) in cells expressing BDNF (El-Bakri et al., 2004; Sohrabji and Lewis, 2006). Experimental evidence suggests that BPA exerts its effects in the hippocampus through estrogenic pathways (Leranth et al., 2008), which can influence NMDAR and intersect with AOPs 12 and 13. Therefore, NMDAR antagonism could be a key event (KE) rather than a molecular initiating event (MIE) when considering the BPA-hippocampal ER effect.

Additionally, neuroinflammation resulting from the disruption of redox homeostasis due to BPA exposure is a plausible pathway for neurodevelopmental alterations. Studies have demonstrated that prenatal exposure to BPA in mice embryos led to increased microglia (brain-resident macrophages) and higher concentrations of inflammatory markers (IL4 and TNF-α) (Takahashi et al., 2018). Moreover, postnatal BPA exposure in adult rats resulted in elevated levels of malondialdehyde, nitric oxide (NO), glutathione peroxidase (GPX), and superoxide dismutase (SOD) (Mohamed Eweda et al., 2021). These findings suggest that BPA-induced neuroinflammation could be another important aspect to consider when assessing its impact on neurodevelopment.

Importantly, some authors identified a potential mediating role of *BDNF* gene methylation at CpG6. Kundakovick and colleagues (2015) found that BPA exposure led to increased DNA methylation at the promoter region IV of the BDNF gene, specifically at CpG1 and CpG2, in highly exposed children (boys but not girls) (Kundakovic et al., 2015). In the same study, offspring from pregnant rats treated orally with BPA displayed alterations in Exon IV of the BDNF gene in the hippocampus and blood, thus finding a correlation between central and peripheral BDNF DNA methylation levels. While findings from the INMA-Granada cohort pilot study no. One aligned with those reported by Kundakovic et al. (2015), there were differences in the associations between BPA exposure and specific CpG sites across the studies. The regulation of BDNF can vary during different developmental stages, which may explain why it was observed significant results with CpG6, but not with CpG1 and CpG2 (Kowiański et al., 2018). Additionally, the longitudinal design of the pilot study enhanced the reliability of the results, thereby validating the use of BDNF as a potential biomarker of neurodevelopmental effects related to BPA exposure.

### 3.2 BDNF as an indicator of behavioral alterations in adolescents exposed to metals

The INMA-Granada cohort pilot study no. 2 was a cross-sectional design focused on the evaluation of four relevant known neurotoxic metals: mercury (Hg), lead (Pb), arsenic (As), and Cadmium (Cd), on serum BDNF protein, BDNF gene DNA methylation, and the behavioral function. Urinary total As concentrations were associated with increased somatic complaints [β, 95% CI: 5.77 (2.08,9.46)]. Increased urinary Cd concentrations were associated with more externalizing problems, such as social problems [β, 95% CI: 4.85 (1.85; 7.85)] or aggressive behavior [β, 95% CI: 3.97 (0.89; 7.05)]. A trend toward lower BDNF protein with increasing As exposure was found in the study, which is consistent with previous findings in adults with chronic exposure to As (Karim et al., 2019). Moreover, it showed strong associations towards higher BDNF DNA methylation at CpG islands 5 and 6 [β, 95% CI: 0.54 (0.11; 0.98); 0.74 (0.09; 1.39), respectively]. In the same line, higher urinary Cd concentrations suggested associations with lower serum BDNF protein levels and significant associations with decreased BDNF DNA methylation at CpGs 2 and 3 [β, 95% CI: 0.39 (−0.67;-0.10); 0.39 (−0.67;-0.10), respectively].

**FIGURE 2:**
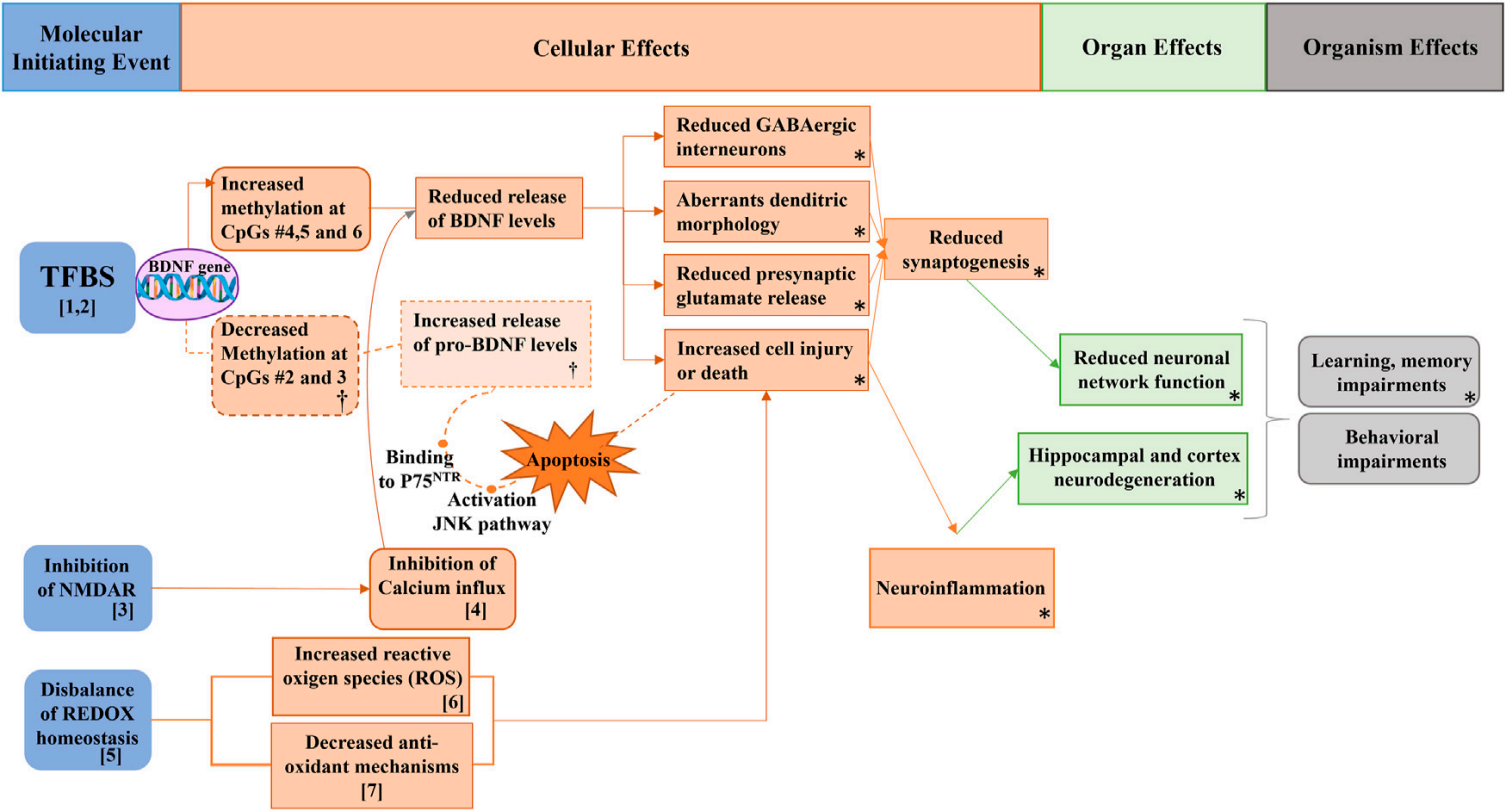
Potential pathways supporting the associations between metals exposure, BDNF levels, and behavioral function in Spanish male adolescents. *Figure modified from* (Rodríguez-Carrillo et al., 2022b). *NMDAR: N-methyl-D-aspartate receptors; BDNF: brain-derived neurotrophic factor; pro-BDNF: immature isoform of BDNF; JNK: c-Jun N-Terminal kinase; TFBS: transcription factor binding sites.* [1,2] As and Cd exposure can interact with TFBS, inhibiting DNA repair mechanisms and thus altering the genetic methylation profile and consequently BDNF levels. [3, 4] Both metals can inhibit NMDAR, inhibiting calcium influx which is crucial for BDNF release. [5,6,7] As and Cd can increase the reactive oxygen species and decrease the anti-oxidant mechanism, thus increasing cell injury and death, leading to neuroinflammation, hippocampal neurodegeneration, and ultimately to learning, memory, and behavioral impairments. (†) makes reference to the hypothesized events triggering P75 pathway through the increment of pro-BDNF levels due to decreased DNA methylation of the BDNF gene. (*) refers to the AOP network developed in Mustieles et al., 2020.

Interestingly, both As and Cd can penetrate the blood-brain barrier (BBB) and inhibit hippocampal NMDAR, as supported by experimental studies (Karri et al., 2016) (Figure 2, number 3). The inhibition of hippocampal NMDAR may lead to reduced BDNF levels, which could explain the observed associations between urinary As and Cd concentrations and changes in serum BDNF protein. Indeed, previous studies involving prenatal exposure to As in rats demonstrated decreased BDNF expression and social isolation-like behavior (Htway et al., 2019). Similarly, male rat pups prenatally exposed to Cd also exhibited decreased BDNF expression and social isolation-like behavior (Mimouna et al., 2018). The hippocampus plays a pivotal role in emotional and stress responses, learning, and memory, all of which are associated with social behavior (Ciranna, 2006). It is especially vulnerable to exogenous chronic stressors; thus any structural or functional alteration could lead to mood disorders development (Zaletel et al., 2017).

**FIGURE 3:**
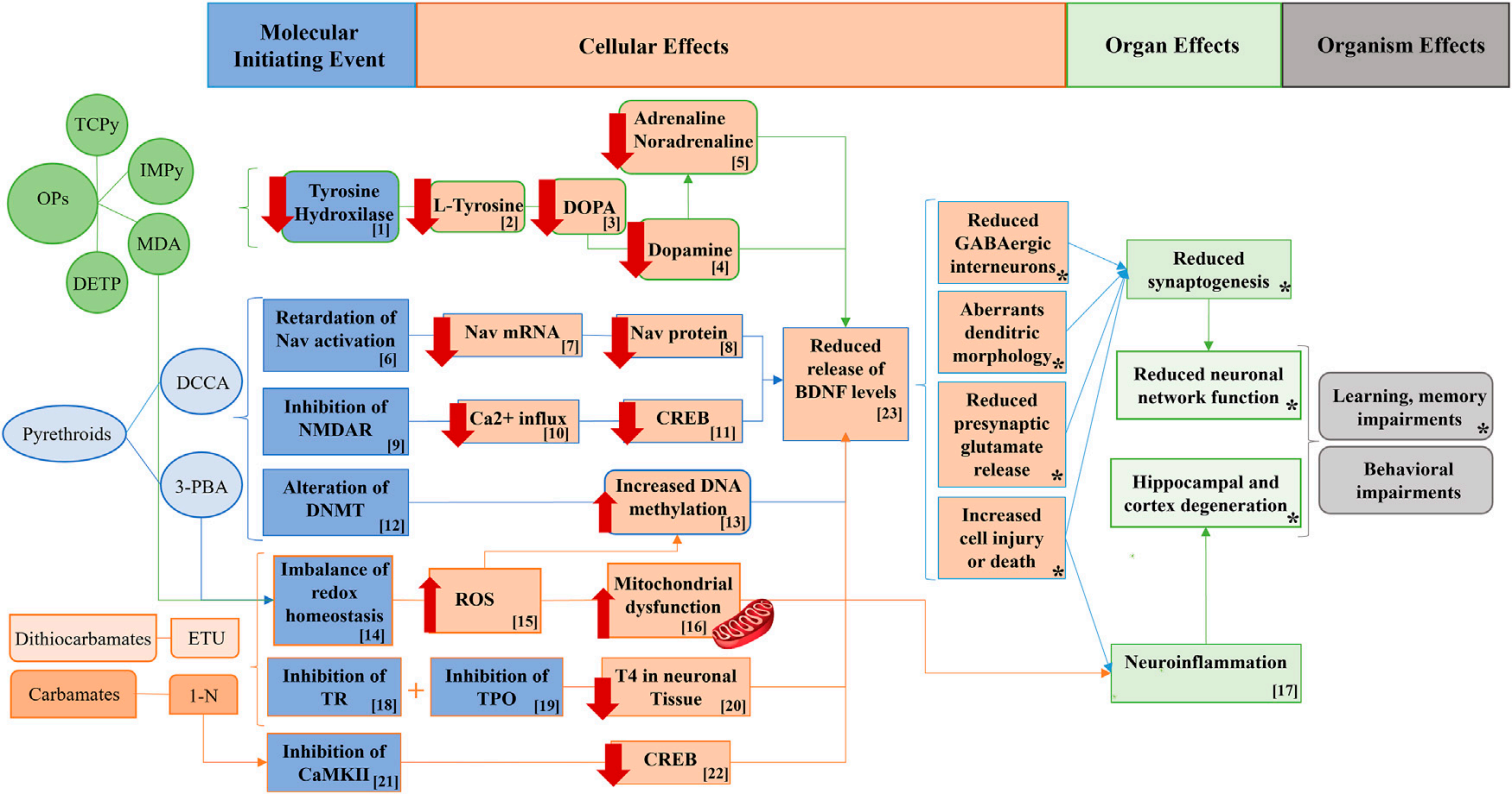
Potential pathways supporting the associations between non-persistent pesticide metabolites, BDNF levels, and behavioral outcomes in adolescent males. *Figure modified from* (Rodríguez-Carrillo et al., 2022a). *OPs: organophosphate pesticides; TCPy: 3,5,6-trichloro-2-pyridinol; IMPy: 2-isopropyl-6-methyl-4-pyrimidinol; MDA: malathion diacid; DETP: diethyl thiophosphate; DCCA: dimethylcyclopropane carboxylic acid; 3-PBA: 3-phenoxybenzoic acid; ETU: ethylene thiourea; 1-N: 1-naphthol; NMDAR: N-methyl D-aspartate receptor; Nav= Sodium channels voltage-dependent; DNMT: DNA methyl transferases; TR: Thyroid receptor; CaMKII: calcium/calmodulin-dependent kinases II; DOPA: dihydroxyphenylalanine; Ca: calcium; ROS: reactive oxygen species; TPO: Thyroid peroxidase; TOH: Tyrosine hydroxylase; CREB: ; T4: ; BDNF: brain-derived neurotrophic factor.* [1] OP pesticides inhibit tyrosine hydroxylase, decreasing L-tyrosine, which is the precursor of the dihydroxyphenylalanine (DOPA), thus decreasing dopamine and therefore adrenaline and noradrenaline. [2, 3] Reduced hippocampal BDNF due to decreased dopaminergic neurotransmitters, potentially leading to behavioral impairments. [4] MDA increases ROS, reducing BDNF levels. [5, 6, 7] Pyrethroids may reduce BDNF levels though a compensation mechanism which reduces Nav expression (Nav mRNA and Nav protein), thus leading to Nav activation retardation. [8, 9] Inhibition of NMDAR by pyrethroids decreased calcium influx, and the activation of CREB, which consequently reduced BDNF levels and thus increasing subsequent behavioral problems in adolescents. [10] Pyrethroids may alter DNMT increasing DNA methylation of BDNF gene and consequently reducing BDNF protein levels. [11, 12] Redox homeostasis imbalance due to pyrethroids exposure may lead to neuroinflammation, with subsequent deleterious effects on the central nervous system. [13, 14, 15, 16 and *] 1-N and ETU are well-documented inhibitors of TR and TPO which would impair the secretion of T4, decreasing BDNF productions and ultimately increasing the probabilities of developing behavioral alterations. [17, 18, 19] CaMKII is inhibited by 1-N, blocking CREB activation and thus the release of BDNF gene transcription. [22, 23, 24] The mitochondrial electron chain may be uncoupled by 1-N and ETU with a subsequent mitochondrial dysfunction, ROS generation and increased neuroinflammation, which may lead to the impairments of behavioral, learning, and memory function. (*) refers to the AOP network developed in Mustieles et al., 2020.

As exposure may impact behavioral function through direct and indirect actions on the *BDNF* gene. It can further alter DNA methylation patterns by potentially interacting with transcription factor binding sites (TFBS) and inhibiting DNA repair mechanisms (Demanelis et al., 2019; Karim et al., 2019) (as depicted in Figure 2, numbers 1 and 2). This could explain the observed increase in BDNF DNA methylation among adolescents with higher urinary As concentrations. The mechanism aligns with experimental findings showing an association between memory deficits and reduced hippocampal BDNF and CREB levels in mice exposed to As (Sun et al., 2015). Moreover, As can exert an indirect effect on BDNF through several pathways. Firstly, it inhibits NMDA receptors, critical for Ca^2+^ influx mechanisms, which leads to reduced BDNF concentrations (Wang et al., 2016a) (Figure 2, number 3 and 4). Secondly, As disrupts oxidative stress homeostasis, elevating reactive oxygen species (ROS) and reducing glutathione (GSH) (Figure 3, number 5, 6, 7) (Karri et al., 2016; Mimouna et al., 2018). This imbalance favors cell injury or death, leading to neuroinflammation and hippocampal cell degeneration, subsequently reducing BDNF expression (Karri et al., 2016) (Figure 3, number 5, 6, 7). Lastly, As can also alter the metabolism of neurotransmitters such as GSH or serotonin, which are essential for BDNF expression and production (Ramos-Chávez et al., 2015; Htway et al., 2019).

According to their findings in mice, (Htway et al., 2019), proposed that developmental arsenic exposure might affect social behavior by modulating serotonin receptors and reducing BDNF concentrations. Findings from this INMA-Granada cohort pilot study no. 2 suggested that exposure to As may be associated with higher BDNF gene DNA methylation. Such alterations could lead to reduced BDNF gene expression levels and protein concentrations, which in turn might contribute to the emergence of behavioral problems.

Cd has also been implicated in neurotoxic activity, affecting various pathways. It can cross the blood-brain barrier, enter the central nervous system (CNS), and disrupt hippocampal membrane function (Kumar et al., 1996; Wang and Du, 2013). In murine studies, Cd exposure inhibited acetylcholine esterase (AchE) and the Na^+^/K^+^-ATP-ase pump, resulting in reduced neuronal activity in pups (Gupta et al., 1991). Additionally, Cd-induced imbalance in redox homeostasis increased neuronal death in rats (Wang and Du, 2013) (Figure 2, numbers 5, 6, 7), and Cd was found to mimic the ubiquitous intracellular ion Ca^2+^, inhibiting its influx pathways (Xu et al., 2011) (Figure 2, number 4).

However, the precise impact of these pathways on hippocampal BDNF expression, either direct or indirect, remains inadequately understood. Nevertheless, some animal studies reported downregulation of BDNF expression after Cd exposure (Kadry and Megeed, 2018; Mimouna et al., 2018), which could support the findings of this second pilot study. The negative association between urinary Cd concentrations and BDNF methylation, together with and its positive associations with higher behavioral problems could be explained with an increase of the pro-BDNF concentrations. This isoform would trigger the cellular apoptosis procedure through its binding to P75 with deleterious consequences for brain development (Zaletel et al., 2017) (Figure 3, †). Similar results were found in Cd-exposed zebrafish, which showed increased BDNF expression together with locomotor alterations (Xia et al., 2020) and in a genome-wide study with *drosophila melanogaster*, which showed that Cd reduced the global DNA methylation (Guan et al., 2019). Given the absence of literature on Cd effects on BDNF secretion patterns, and since pro-BDNF was not measured in the pilot study, further research is warranted to refute or verify this hypothesis (Rodríguez-Carrillo et al., 2022b).

### 3.3 BDNF as a potential mediator of behavioral function in adolescents exposed to non-persistent pesticides

In the INMA-Granada cohort pilot study no. 3, a cross-sectional design, eight non-persistent pesticide metabolites and their mixture effect were assessed, which included the following compounds. Five organophosphate (OP) metabolites: 3,5,6-trichloro-2-pyridinol (TCPy), 2-isopropyl-6-methyl-4-pyrimidinol (IMPy), and malathion diacid (MDA), which are specific metabolites of the OP insecticides chlorpyrifos/chlorpyrifos-methyl, diazinon, and malathion, respectively; dialkyl phosphates diethyl thiophosphate (DETP) and diethyl dithiophosphate (DEDTP), which are non-specific metabolites of OP insecticides. Two pyrethroid metabolites: 3-phenoxybenzoic acid (3-PBA), which is a common metabolite to several pyrethroids, and dimethylcyclopropane carboxylic acid (DCCA) (sum of cis and trans isomers), which are metabolites of cypermethrin, cyfluthrin, or permethrin. Last, the major metabolite of ethylene-bis-dithiocarbamate (EBDC) fungicides, ethylene thiourea (ETU), and a metabolite of the carbamate insecticide carbaryl, 1-naphthol (1-N) were measured. Additionally, the sum of organophosphate (ΣOPs) and pyrethroid (ΣPYR) metabolites was calculated and the overall mixture effect was also considered. Organophosphate and pyrethroid compounds are the most widely used pesticides worldwide. They interfere with the function of the nervous system of insects, and to a lesser degree with that of mammals, therefore raising concerns for human health (Oulhote and Bouchard, 2013).

Urinary IMPy and DETP showed associations with lower serum BDNF protein [β_T3_, 95% CI: −4.29 (−8.33,-0.25); −3.82 (−8.25,0.61), respectively], while MDA was associated with lower serum BDNF levels [β_T3_, 95% CI: −6.74 (−11.38,-2.10] and higher BDNF DNA methylation percentage, particularly at CpG2 [β_T2_, 95% CI: 0.26 (0.04,0.46)]. Additionally, ΣOPs showed a strong and dose-dependent association with lower serum BDNF levels [β_T3_, 95% CI: −5.89 (−10.19,-1.58)]. OP insecticides are known neurotoxic chemicals due to their action as inhibitors of acetylcholinesterase (AChE) (Richendrfer and Creton, 2015). Nevertheless, other non-cholinergic pathways are also plausible. The dopaminergic system is one such non-cholinergic pathway that plays a vital role in BDNF regulation through tyrosine hydroxylase (TOH) inhibition (Küppers and Beyer, 2001) (Figure 3, number 1). TOH is an enzyme responsible for synthesizing L-tyrosine, the precursor of dihydroxyphenylalanine (DOPA), which eventually leads to dopamine production (Shin et al., 2001) (Figure 3, numbers 1, 2, 3, 4, 5). Experimental evidence has shown that OP pesticides such as diazinon and malathion can inhibit TOH, resulting in reduced dopamine levels and subsequent behavioral alterations, such as increased anxiety in different organisms (Shin et al., 2001; Ahmed et al., 2017).

Furthermore, exposure to chlorpyrifos has been found to downregulate BDNF expression and decrease the cholinergic system in zebrafish, leading to increased impulsive behavior (Perez-Fernandez et al., 2020) (Figure 3, number 23). This may support the observed association with Rule-Breaking behavior [IMPy = β_T3_, 95% CI: 3.76 (1.06,6.45)], from which serum BDNF protein suggested a small mediation effect of 7.6% [β, 95% CI: 0.11 (−0.07,0.57)]. Additionally, a study on malathion-exposed rats showed increased reactive oxidative stress species (ROS) and reduced hippocampal BDNF expression, providing further mechanistic support for the observed relationship between OP pesticide exposure and decreased BDNF levels (Ardebili Dorri et al., 2015) (Figure 3, number 14, 15).

Regarding pyrethroids, increased urinary concentrations of 3-PBA were associated with increased BDNF gene DNA methylation at CpGs 4 and 6 [β, 95% CI: 0.65 (0.03,1.26); 0.57 (0.02,1.12), respectively]. There are potential mechanisms underpinning the observed relationship between these outcomes. Firstly, permethrin has been shown to interact with DNA methyltransferases (DNMT), leading to alterations in DNA methylation patterns *in vivo* (Bordoni et al., 2015) (Figure 3; numbers 12, 13). Second, NMDAR inhibition following exposure to permethrin and deltamethrin in both *in vivo* and *in vitro* models can lead to downregulation of CREB, a key regulator of BDNF expression, resulting in hippocampal BDNF mRNA repression (Imamura et al., 2000; Zhang et al., 2018) (Figure 3; numbers 9, 10, 11). Third, cypermethrin and bifenthrin exposure has been associated with increased reactive oxygen species (ROS), leading to neuroinflammation and long-lasting behavioral impairments in murine models (Nasuti et al., 2007; Gargouri et al., 2018) (Figure 3, numbers 14, 15, 17). Last, pyrethroids may affect sodium channel voltage-dependent (Nav) activation, resulting in downregulation of Nav expression as a compensatory mechanism. This downregulation has been linked to decreased BDNF expression in studies involving deltamethrin-exposed mice (Imamura et al., 2006; Magby and Richardson, 2017) (Figure 3; numbers6, 7, 8, and 23).

Concerning carbamate and dithiocarbamate metabolites, 1-N was associated with lower BDNF serum levels [β, 95% CI: −3.91 (−7.35,-0.46)], whereas ETU was associated with more social problems [β_T2_, 95% CI:3.18 (0.64,5.71)] and higher BDNF gene DNA methylation at several CpGs, concretely 2 and 3 [β_T3_, 95% CI: 0.27 (0.05,0.49); 0.41 (0.15,0.67), respectively]. Focusing on the observed associations between 1-N, ETU, and BDNF biomarkers, there are some mechanisms supporting these findings. Firstly, murine models exposed to 1-N and ETU exhibited inhibition of thyroid receptor (TR) and thyroid peroxidase (TPO), resulting in compromised thyroid hormone secretion and a decrease in hippocampal BDNF synthesis (Figure 3; numbers 18, 19, 20, 23). These effects ultimately contribute to neurodevelopmental alterations (Marinovich et al., 1997; Sun et al., 2008; Maranghi et al., 2013; Shafiee et al., 2016). Secondly, in both *in vivo* and *in vitro* models, exposure to 1-N was found to inhibit CaMKII, impairing CREB phosphorylation and the subsequent transcription of BDNF. This leads to reduced BDNF-mediated neurite growth (Saito et al., 2013; Lee et al., 2015; Islam et al., 2019) (Figure 3; numbers 21, 22, and 23). Finally, 1-N and ETU have been shown to disrupt the mitochondrial electron transport chain, inducing mitochondrial dysfunction, generating reactive oxygen species (ROS), and ultimately triggering neuroinflammation, which contributes to subsequent behavioral impairments (Domico et al., 2007; Gupta et al., 2007; Bjørling-Poulsen et al., 2008; Muthaiah Venkitasamy et al., 2013; He et al., 2020) (Figure 3; numbers 15, 16, 17).

Importantly, this pilot study no. 3 was the first epidemiological study assessing the mixture effect of non-persistent pesticide exposure on BDNF and the behavioral function. Results showed that this mixture was associated with higher withdrawn [β, *p*-value: 2.71, 0.05] and social [β, *p*-value: 4.27, <0.01] problems as well as higher BDNF DNA methylation at CpG3 [β, *p*-value: 2.26, <0.03], 6 [β, *p*-value: 0.49, 0.07], and total CpGs [β, *p*-value: 0.27, 0.03]. Interestingly, the higher contributor to the mixture effect was MDA, although when studied individually, it was not associated either with behavioral function or with any BDNF biomarker. It was hypothesized that this was due to an additive effect, which may be from the OPs included in the mixture (IMPy), which may share the same modes of action. There is little information regarding the mixture effect of pesticides on BDNF. Only an *in vitro* study found increased BDNF mRNA using a mixture that included deltamethrin, cyfluthrin, and chlorpyrifos (Özdemir et al., 2018). Using a computation mixture approach, this pilot study showed the importance of evaluating the combined effect of chemical pollutants.
