# Supplementary material for: Brain-derived neurotrophic factor (BDNF): an effect biomarker of neurodevelopment in human biomonitoring programs
Source: Front Toxicol. 2024 Jan 10;5:1319788. doi: 10.3389/ftox.2023.1319788 (PMC10806109; doi:10.3389/ftox.2023.1319788)
Supplement: Supplementary file 1 [file DataSheet1.pdf]

SUPPLEMENTARY MATERIAL

Brain-Derived Neurotrophic Factor (BDNF): an effect biomarker of neurodevelopment in human biomonitoring programs

Andrea Rodríguez-Carrillo, Veerle J Verheyen, Alexander L.N. van Nuijs, Mariana F. Fernández, Sylvie Remy

Table S1. The National Institutes of Health (NIH) quality assessment tool for observational cohort and cross-sectional studies (n=22)

Author	Study type	Selection				Comparability		Outcome		Total Score
		1	2	3	4	5	6	7	8	
(Wang et al., 2023)	Longitudinal	1	1	1	1	2	1	1	1	9
(Huang et al., 2022)	Longitudinal	1	1	1	1	2	1	1	1	9
(Sarzo et al., 2022)	Longitudinal	1	1	1	1	2	1	1	1	9
(Yu et al., 2021)	Longitudinal	1	1	1	1	2	1	1	0	8
(Zhou et al., 2019b)	Longitudinal	1	1	1	1	2	1	1	1	9
(Wang et al., 2016b)	Longitudinal	1	1	1	1	2	1	1	0	8
(Saenen et al., 2015)	Longitudinal	1	1	1	1	2	1	1	1	9
(Julvez et al., 2013)	Longitudinal	1	1	1	1	2	1	1	1	9
(Song et al., 2023)	Cross-sectional	0	1	1	1	2	1	0	1	7
(Song et al., 2022)	Cross-sectional	1	1	1	1	2	1	0	1	7
(Zhou et al., 2021)	Cross-sectional	1	1	1	1	2	1	0	1	8
(Malavika et al., 2021)	Cross-sectional	1	1	1	1	1	1	0	1	7
(Hogervorst et al., 2021)	Cross-sectional	1	1	1	1	2	1	0	1	8
(Lozano et al., 2021)	Cross-sectional	1	1	1	1	2	1	0	1	8
(Zhou et al., 2019)	Cross-sectional	1	1	1	1	2	1	0	1	8
(Zaw and Taneepanichskul, 2019)	Cross-sectional	1	1	1	1	2	1	0	1	8
(Karim et al., 2019)	Cross-sectional	1	1	1	1	2	1	0	1	8
(Zhang et al., 2017)	Cross-sectional	1	1	1	1	1	1	0	1	7
(Perera et al., 2015)	Cross-sectional	1	1	1	1	2	1	0	1	8
(Zhu et al., 2022)	Case-Control	1	1	1	1	2	1	1	1	9

(Yang et al., 2016)	Case-Control	1	1	1	1	2	1	1	1	9
(Zou et al., 2014)	Case-Control	1	1	1	1	2	1	1	1	9

Questions applied for cohort studies: 1. Representativeness of the exposed cohort [a) truly representative of the average children/adolescents/pregnant women/adults in the community*; b) somewhat representative of the average children/adolescents/pregnant women/adults in the community*; c) selected group of users e.g., nurses, volunteers; d) no description of the derivation of the cohort]. 2. Selection of the non-exposed cohort [a) drawn from the same community as the exposed cohort*; b) drawn from a different source; c) no description of the derivation of the non-exposed cohort]. 3. Ascertainment of exposure [a) secure record (e.g., surgical records)/lab measurement*; b) structured interview*; c) written self-report; d) no description]. 4. Demonstration that outcome of interest was not present at start of study [a) yes, it was healthy population*; b) no]. 5. Comparability of cohorts based on the design or analysis [a) study controls for age and sex*; b) study controls for any additional factor* (e.g., body mass index, maternal education, alcohol consumption, or smoking)]. 6. Assessment of outcome [a) independent blind assessment*; b) record linkage; c) self-report; d) no description]. 7. Was follow-up long enough for outcomes to occur [a) yes* (1 years minimum); b) no]. 8. Adequacy of follow up of cohorts [a) complete follow up*; b) subjects lost to follow up unlikely to introduce bias/small number lost (35 %); c) follow up rate above 35 % and no description of those lost; d) no statement].
Questions applied for case-control studies: 1. Is the case definition adequate? [a) yes, with independent validation*; b) yes, e.g., record linkage or based on self-reports; c) no description]. 2. Representativeness of the cases [a) consecutive or obviously representative series of cases*; b) potential for selection biases or not stated]. 3. Selection of Controls [a) community controls*; b) hospital controls; c) no description]. 4. Definition of Controls [a) no history of disease (endpoint)*; b) no description of source]. 5. Comparability of cases and controls based on the design or analysis [a) study controls for the most important factor*; b) study controls for any additional factor*; 6. Ascertainment of exposure [a) secure record (e.g., surgical records)*; b) structured interview where blind to case/control status*; c) interview not blinded to case/control status; d) written self-report or medical record only; e) no description]. 7. Same method of ascertainment for cases and controls [a) yes*; b) no]. 8. Non-response rate [a) same rate for both groups*; b) non respondents described; c) rate different and no designation].
(*) One point is granted if that option is selected. A study can be awarded a maximum of one point for each numbered item within Selection and Exposure categories; a maximum of two stars can be given for Comparability (question 5).

Table S2. Frequencies of participants' age-range of included studies (n=22 studies).

	**Newborns/Mother-son pairs**											**Children**					**Adults**						**Total sample size**
**No. study**	1	9	3	11	4	12	5	6	16	13	22	19	10	21	15	20	2	7	8	14	17	18	
**n per study**	711	541	725	443	227	561	300	90	505	108	466	413	72	403	118	843	34	34	34	693	24	348	7693
**Total**	4211											2315					1167						
**%**	54.74											30.09					15.17						100
